# Friction Durability of Extremely Thin Diamond-Like Carbon Films at High Temperature

**DOI:** 10.3390/ma10020159

**Published:** 2017-02-10

**Authors:** Shojiro Miyake, Shota Suzuki, Masatoshi Miyake

**Affiliations:** 1 Nippon Institute of Technology, 4-1 Gakuendai, Miyashiro-machi, Saitama 345-8501, Japan; suzuki1992umare@gmail.com; 2 Faculty of Literature, Nishogakusha University, 6-16, Sanbancho, Chiyoda-ku, Tokyo 102-8336, Japan; toshimiya1@yahoo.co.jp

**Keywords:** friction durability, high temperature, extremely thin film, diamond-like carbon (DLC), nano-friction

## Abstract

To clarify the friction durability, both during and after the high-temperature heating of nanometer-thick diamond-like carbon (DLC) films, deposited using filtered cathodic vacuum arc (FCVA) and plasma chemical vapor deposition (P-CVD) methods, the dependence of the friction coefficient on the load and sliding cycles of the DLC films, were evaluated. Cluster-I consisted of a low friction area in which the DLC film was effective, while cluster-II consisted of a high friction area in which the lubricating effect of the DLC film was lost. The friction durability of the films was evaluated by statistical cluster analysis. Extremely thin FCVA-DLC films exhibited an excellent wear resistance at room temperature, but their friction durability was decreased at high temperatures. In contrast, the durability of the P-CVD-DLC films was increased at high temperatures when compared with that observed at room temperature. This inverse dependence on temperature corresponded to the nano-friction results obtained by atomic force microscopy. The decrease in the friction durability of the FCVA-DLC films at high temperatures, was caused by a complex effect of temperature and friction. The tribochemical reaction produced by the P-CVD-DLC films reduced their friction coefficient, increasing their durability at high temperatures.

## 1. Introduction

The development of advanced electronic data storage devices has been realized in recent years. In high-density recording magnetic disk drives, diamond-like carbon (DLC) film is usually applied to magnetic recording head-disk interfaces, as a protective film [1,2]. Therefore, improving the tribological durability of magnetic head-disk interfaces is important for the future of the rapidly growing magnetic recording industry. Extremely thin DLC protective films on magnetic heads and disks play an important role in realizing higher-reliability magnetic storage devices, and the methods used to deposit these extremely thin protective films have become important [1,2,3,4,5].

To obtain higher-density magnetic storage, the magnetic space at the magnetic head-disk interface must be decreased, which in turn, requires a reduction in the thickness of the DLC protective film. An extremely thin layer of DLC protective film, typically thinner than 2 nm, is usually applied to the magnetic layer. However, it is difficult to maintain mechanical durability as the thickness of the deposited DLC film is decreased [6,7]. Thus, the durability of extremely thin (about 1 nm thick) DLC film, subjected to friction and wear, should be evaluated. Additionally, heat-assisted magnetic recording (HAMR) technology has been developed to further realize high-density storage. In this technology, data are magnetically recorded on high-stability magnetic media, using thermal assistance to heat the material [8,9]. Therefore, it is also necessary to clarify the high temperature durability of the extremely thin DLC films used as protective films on HAMR magnetic disks and heads [10,11,12]. Plasma chemical vapor deposition (P-CVD) is currently used for the deposition of thin DLC films on magnetic disks, but filtered cathodic vacuum arc (FCVA) ta-C (tetrahedral amorphous carbon) thin films, are expected to be applied to magnetic disks in the future, owing to their higher hardness and density values [13,14,15,16,17,18]. FCVA-DLC films have a greater potential for resistance to high temperatures [11,12] and plastic deformation, than that of P-CVD-DLC films [19,20].

In our former studies, the friction coefficient and wear depth of an FCVA-DLC film were found to be initially low, before increasing rapidly at critical load, whereas the values for a P-CVD-DLC film increased gradually under nano scratch tests, even at low load. These results were deduced to have been caused by the higher hardness and brittleness of FCVA-DLC films, when compared to those of P-CVD-DLC films [21]. Protuberance and groove formation in extremely thin DLC films on Si substrates, caused by diamond tip sliding, was also evaluated by atomic force microscopy [22].

Moreover, we have previously evaluated the nanotribological properties of extremely thin DLC films at high temperatures, using atomic force microscopy with and without vibration. At room temperature, FCVA-DLC films showed superior nanowear resistance to that of P-CVD-DLC films. However, at high temperatures in a vacuum, the wear rapidly increased [23]. We also studied the dependence of the durability of FCVA-DLC and P-CVD-DLC films on their thickness, using four kinds of friction tests to evaluate the superior durability of extremely thin FCVA-DLC film at room temperature [24].

In this study, to clarify the friction durability of extremely thin DLC films at high temperatures, the dependence of the friction properties of DLC films, deposited using the FCVA and P-CVD-DLC methods, on the load and reciprocating cycle number, was evaluated at high temperatures. The obtained friction coefficients were classified into two clusters by statistical cluster analysis, representing high friction and low friction regions, in order to evaluate the friction durability boundary conditions. To clarify the process of DLC degradation caused by sliding, the dependence of the friction coefficient on the load and temperature was analyzed by nano-friction tests, using atomic force microscopy.

## 2. Materials and Methods

### 2.1. Evaluated Diamond-Like Carbon Films

Extremely thin protective DLC films with thicknesses of 0.03, 0.1, 0.2, 0.3, 0.4, 0.6, 0.8, 1.0, 1.5, 2.0, 5.0, and 100 nm, were deposited on the surface of silicon wafers (Si (100)) using the FCVA and P-CVD-DLC methods [24]. To evaluate the dependence of durability on the film thickness at high temperatures, we deposited DLC films of various thicknesses, including minimally thick initial layers, by changing the deposition time. For example, the 0.03- and 0.1-nm thick layers were thinner than the C-C covalent bond length (0.154 nm), and thus, a uniform film was not formed for these samples. However, we measured the friction durability of the 0.03- and 0.1-nm thick initial layer of the DLC film, to clarify the critical thickness at which durability was improved. The difference in the surface roughness between the films deposited by FCVA and P-CVD methods, was negligible. The roughness of each of these surfaces was as low as 0.09–0.2 nm Sa and was similar to that of the Si substrate [24]. Transmission electron microscopy (TEM, HD-2000, Hitachi High-Technologies, Tokyo, Japan) was used to determine the thickness of each DLC film. 

### 2.2. Load Increase-and-Decrease Type Friction Test

**Figure 1:**
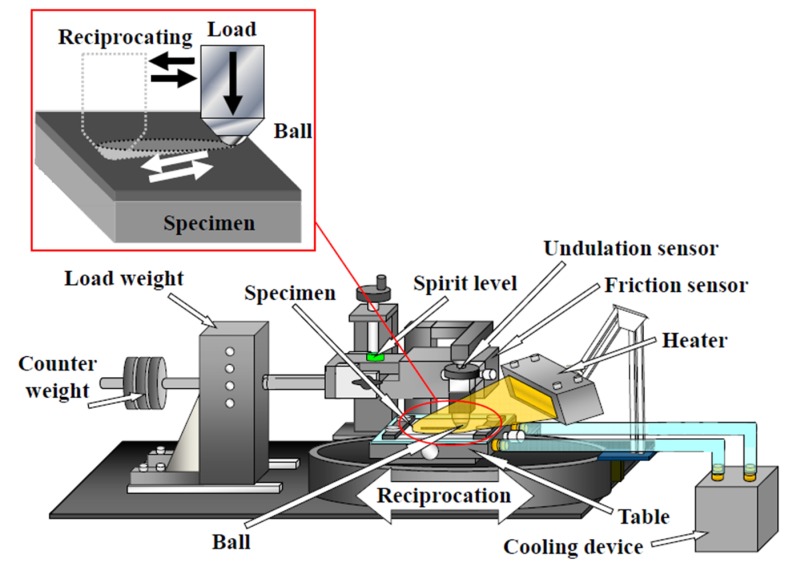
Load increase and decrease reciprocation friction test.

Load increase and decrease friction tests were performed, to evaluate the dependence of the tribological properties of the extremely thin DLC films on temperature, as shown in Figure 1. The surface of each specimen was heated using a near-infrared condensed light beam. The surface temperature was measured by a thermocouple fixed to the specimen and was controlled to under ±3 °C during the friction test. A 2.0-mm-diameter Al_2_O_3_-TiC ball was used as the opposite specimen, without lubrication. The friction properties of the DLC film-coated Si (100) substrates were evaluated with and without infrared beam irradiation at room temperature (nearly 20 °C), 100, and 200 °C. The friction test was started after the temperature of the specimen had stabilized at the test temperature. The test specimen was fixed on a reciprocating stage. Load increase and decrease on the opposite ball attachment were performed gradually, as the stage was moved reciprocally, and the resulting friction force was measured with a strain gauge, from the minimum load to the maximum load. The load was increased and decreased between 0.05 and 0.5 N in each cycle (Hertzian stress; 0.45–1.07 GPa), to evaluate the friction dependencies on load and surface degradation [25]. To clarify the degradation mechanism of the DLC films at high temperatures, friction tests were also performed at room temperature, after heating at 100, 200, 300, and 500 °C, for 3 h.

### 2.3. Nano-Friction Test

**Figure 2:**
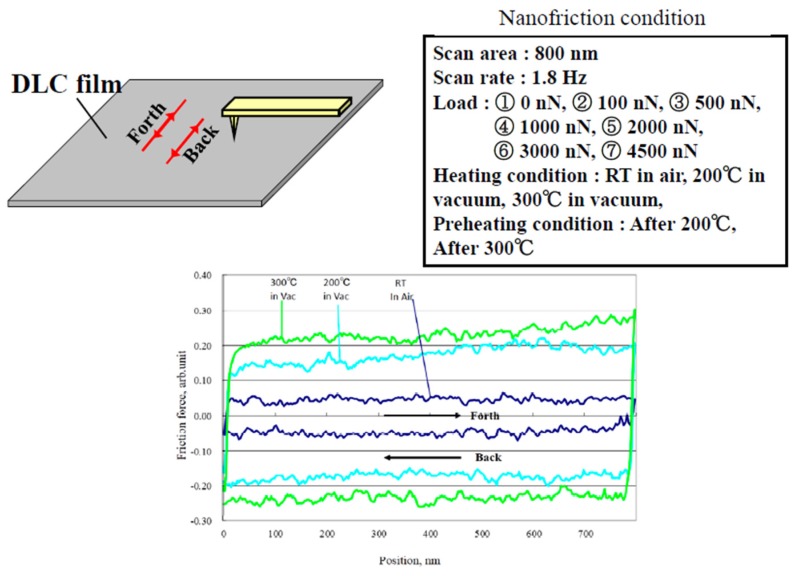
Nano-friction test.

To evaluate the change in the durability of the DLC films at high temperature, the dependence of their nano-friction properties on the applied load was evaluated using environmentally controlled atomic force microscopy (AFM) [23], as shown in Figure 2. No changes in the structure or profile of the DLC films were clear from our surface analysis, based on Fourier transform infrared and X-ray photoelectron spectroscopy measurements of a low friction trace of the p-CVD DLC film. Thus, we tried to evaluate the nano-friction properties by AFM, and compared the macroscopic friction durability and nano-friction properties, and their dependence on the surface temperature. The friction curves of the FCVA-DLC and P-CVD-DLC films were measured under various conditions. The relative friction force was defined as half of the difference between the forward and backward directions of the friction curves. To avoid damage and degradation of the electronic parts of the AFM, the friction tests at high temperatures were performed in a vacuum. First, the sample was placed in the vacuum chamber and a nano-friction test was performed at room temperature (nearly 20 °C), in air atmosphere. Then, the chamber was evacuated using rotary and turbo molecular pumps, to achieve a vacuum of 6 × 10^−5^ Pa. The nano-friction test was then performed again at room temperature, after which the specimen was heated from room temperature, to 100, 200, and 300 °C in the vacuum, and the tests were carried out at each temperature, at a different position on the sample. The nano-friction test was performed by sliding the tip, a ca. 150-nm-radius diamond tip fixed on a cantilever, under the load, and scanning over a 1000-nm length. The test load was changed from 0 to 4500 nN (Hertz stress GPa). Each nano-friction test was performed more than three times, and the average and typical data were considered. Finally, to evaluate the degradation of the DLC films at high temperatures, the nano-friction test was also performed in air at room temperature, after the specimen had been heated to 100–300 °C.

## 3. Experimental Results and Discussion

### 3.1. Macroscopic Tribological Properties at High Temperature Evaluated by Load Increase-and-Decrease Type Friction Test

#### 3.1.1. Dependence of Friction Properties on Reciprocating Cycle Number and Applied Load

**Figure 3:**
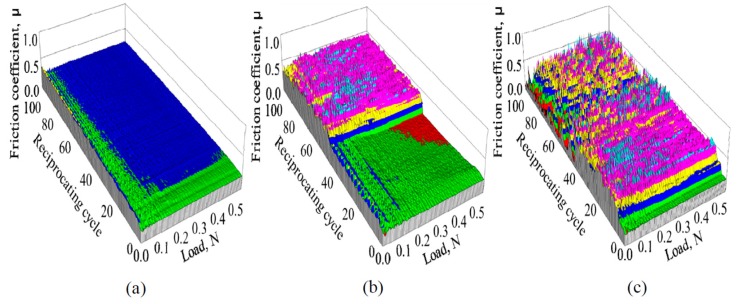
Friction properties of 0.4 nm FCVA-DLC films at various temperatures. (**a**) RT; (**b**) 100 °C; (**c**) 200 °C.

The friction coefficients of 0.4 nm-thick FCVA-DLC film at room temperature (a); 100 °C (b); and 200 °C (c), are shown in Figure 3. The friction coefficient was stable at nearly 0.15 at room temperature, over 100 cycles. However, when the surface was heated at 100 or 200 °C, the friction coefficient increased rapidly over a short number of reciprocating cycles. The friction coefficient of the 0.4 nm-thick FCVA-DLC film increased rapidly after about 57 cycles. This rapid increase occurred at nearly the same number of cycles as that observed without low load conditions. For these friction increase conditions, damage was clearly observed. It was deduced that the friction coefficient increased at a similar reciprocating cycle number because damage first occurred at high load, and then progressed from the high load area, to the lower load side. At room temperature and 100 °C, the 5.0-nm-thick FCVA-DLC film showed the best durability. However, at 200 °C, the friction coefficient increased at nearly 65 cycles.

**Figure 4:**
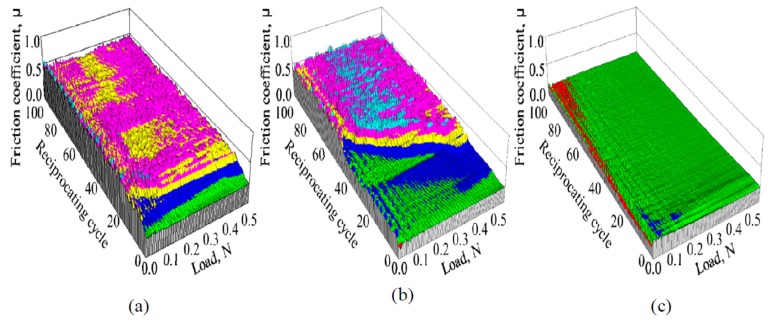
Friction properties of 0.4 nm P-CVD-DLC films at various temperatures. (**a**) RT; (**b**) 100 °C; (**c**) 200 °C.

The friction coefficients measured for the 0.4-nm-thick P-CVD-DLC films are shown in Figure 4. At room temperature, the friction coefficient of the 0.4-nm-thick P-CVD-DLC film increased with the reciprocating cycle number. However, at 100 and 200 °C, the friction coefficient was low until much later reciprocating cycles. The cycle number at which the friction coefficient started to increase at 100 °C changed with the load, with the result obtained at a lower load, different to that observed at room temperature. At 200 °C the friction coefficient of the P-CVD-DLC film was low and stable, as shown in Figure 4c. At both 100 and 200 °C, the friction coefficient of the 5-nm-thick P-CVD-DLC film was stable and showed a longer durability than when at room temperature. Lubricative low friction properties were observed at high temperatures.

#### 3.1.2. Evaluation of Durability by Cluster Analysis of the Friction Coefficient

**Figure 5:**
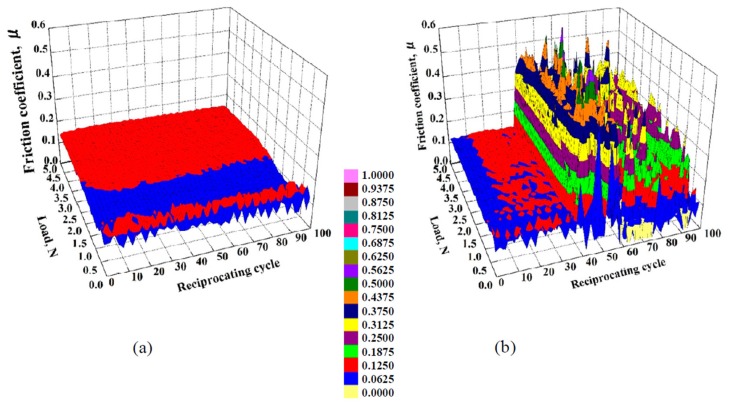
Friction properties of 1.0 nm FCVA-DLC films at various temperatures. (**a**) RT; (**b**) 200 °C.

**Figure 6:**
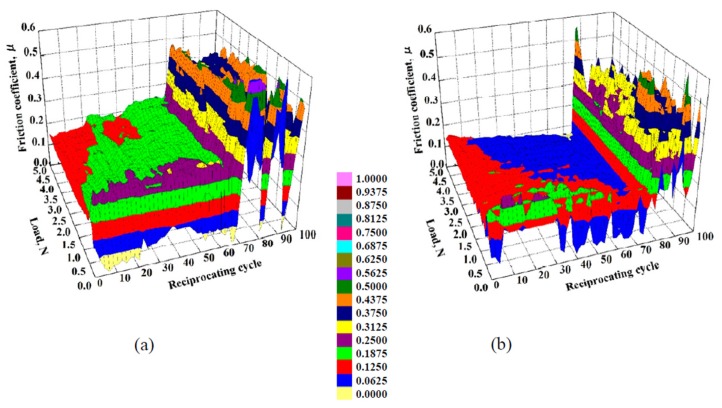
Friction properties of 1.0 nm P-CVD-DLC films at various temperatures. (**a**) RT; (**b**) 200 °C.

**Figure 7:**
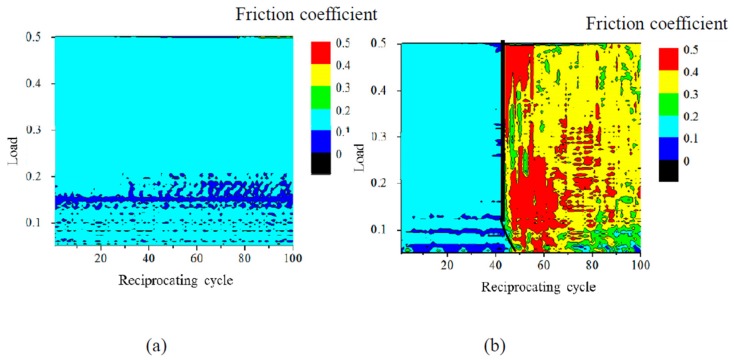
Contour plot and overhead view of cluster analysis data for friction coefficient of FCVA-DLC film. (**a**) RT; (**b**) 200 °C.

The dependence of the friction coefficient of 1-nm-thick FCVA-DLC and P-CVD-DLC films on the reciprocating cycle number and load, are shown in Figure 5 and Figure 6, respectively. The 1.0-nm-thick FCVA-DLC film showed excellent durability and maintained a low friction coefficient at room temperature. At 200 °C, however, the friction coefficient of the FCVA-DLC film gradually increased, and then rapidly increased after a low number of sliding cycles, as shown in Figure 5. In contrast, the friction coefficient of the P-CVD-DLC film tended to increase at room temperature, and only increased rapidly after a certain number of cycles as shown in Figure 6. At 200 °C, the friction coefficient of the P-CVD-DLC film decreased, and its durability cycle increased. Clustering analysis of the dependence of the friction coefficient on the load and number of reciprocating cycles obtained from the friction test was performed with the statistical analysis software R [26]. A hierarchical clustering technique, the k-means method, was applied in the analysis, to evaluate the durability of the DLC films. The dependence of the friction coefficient on the load and number of reciprocating cycles was classified into two clusters; cluster-I represented a low friction region in which the DLC film was effective, and cluster-II represented a high friction region where the lubricating effect of the DLC film was lost. Figure 7 shows a contour plot and an overhead view of the cluster analysis data for the FCVA-DLC film. Table 1 shows the average and maximum friction coefficients of cluster-I, and the average and minimum friction coefficients of cluster-II. There was a certain boundary value at which the effectiveness of the DLC film was lost. When the friction coefficient exceeded that value, the region changed from cluster-I, to cluster-II. Thus, the friction durability cycle and load could be evaluated from the boundary conditions of these clusters. The boundary friction coefficient values were obtained by averaging these maximum and minimum values. The durability of the films was evaluated from the average number of reciprocating cycles endured by the cluster analysis. At room temperature, the friction coefficient of the FCVA-DLC film remained low, with a value of nearly 0.13. At high temperatures, the friction coefficient was also initially as low as 0.13 in cluster-I, however, the number of cycles before the friction coefficient changed from cluster-I to cluster-II, was as short as about 50 cycles. The average friction coefficient of the FCVA-DLC film was 0.38, falling in cluster-II. 

**Figure 8:**
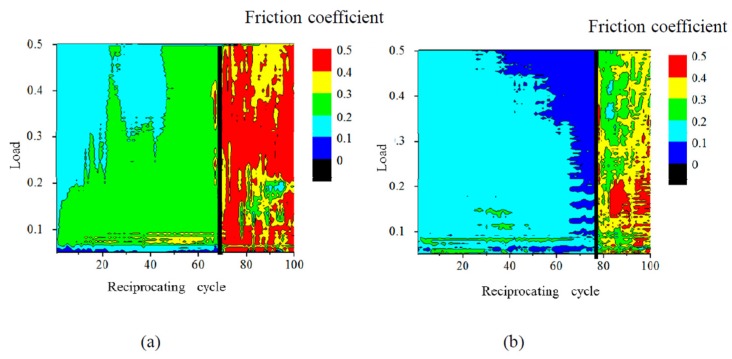
Contour plot and overhead view of cluster analysis data for friction coefficient of P-CVD-DLC film. (**a**) RT; (**b**) 200 °C.

Figure 8 shows the cluster analysis data for 1.0-nm-thick P-CVD-DLC film after the sliding test. Table 1 shows the average and boundary friction coefficients of each cluster. The average friction coefficient of the P-CVD-DLC film in cluster-I at room temperature, was higher than 0.21, and thus greater than that of the FCVA-DLC film. The friction coefficient increased gradually. The number of cycles after which the friction coefficient increased rapidly was about 70 cycles at room temperature. At 200 °C, the cluster-I average friction coefficient of the P-CVD-DLC film was lower than that at room temperature, producing values of 0.129 and 0.214, respectively. The friction coefficient increased rapidly after nearly 80 cycles, indicating that the film was durable for a longer time than when it was at room temperature. 

#### 3.1.3. Dependence of Durability on Film Thickness and Surface Temperature

**Figure 9:**
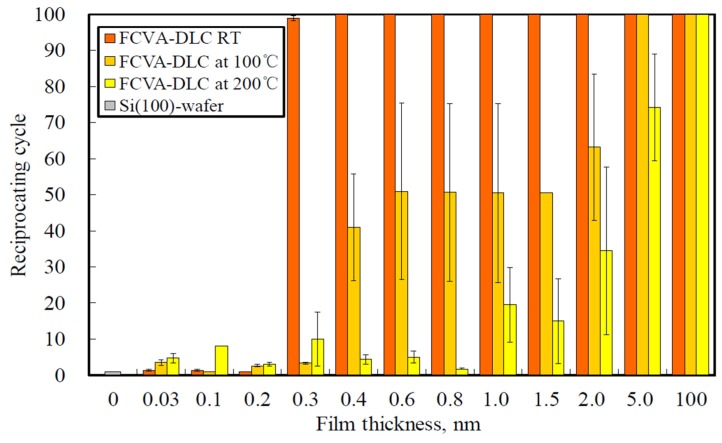
Endurance reciprocating cycle of FCVA-DLC films at various temperatures.

Figure 9 shows the dependence of the durability of the FCVA-DLC films on their thickness, as evaluated from the cluster analysis. The average reciprocating cycle number at which the friction coefficient began to rapidly increase, was evaluated. At room temperature, the durability of the FCVA-DLC films was superior to that of the P-CVD-DLC films. The durability of the 0.4-nm or thicker FCVA-DLC films showed no decline over the number of test cycles. However, at high temperatures, the friction durability of the sub-2-nm-thick FCVA-DLC films decreased. At 100 and 200 °C, the friction durability of the thin FCVA-DLC films became remarkably short. Only the 100-nm-thick FCVA-DLC film remained durable for over 100 cycles, at both 100 and 200 °C. The cycle endurance of the thin FCVA-DLC films decreased when at a high temperature, both at 100 and 200 °C. These increases in the friction coefficient, and decreases in the friction durability of the extremely thin FCVA-DLC films at high temperatures, were clearly evaluated. 

**Figure 10:**
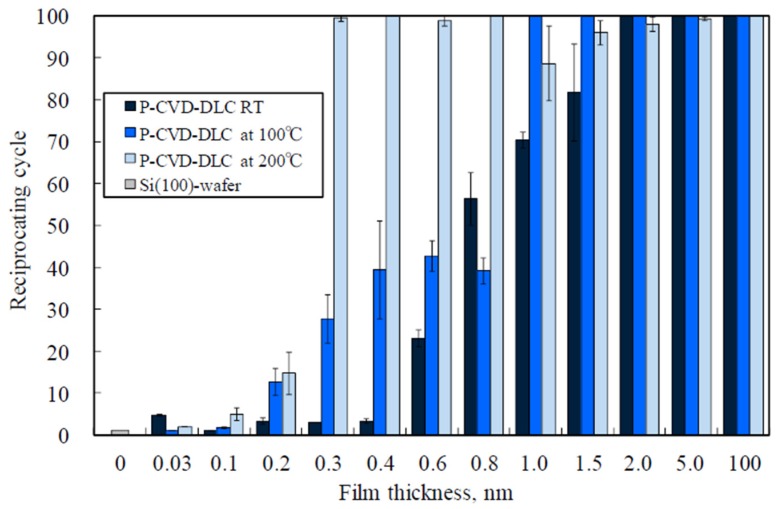
Endurance reciprocating cycle of P-CVD-DLC films at various temperatures.

Figure 10 shows the dependence of the durability of the P-CVD-DLC films on their thickness at room temperature, 100, and 200 °C. At room temperature, the films were durable for a shorter time than the FCVA-DLC films. The number of cycles that the films endured increased with the film thickness. At room temperature, the lubricating effect of the P-CVD-DLC film was easily lost through the reciprocating sliding. However, at 200 °C, the friction durability of the P-CVD-DLC films increased, even when the film was as thin as 0.2–0.8 nm. The friction durability of the 1.0-nm-thick P-CVD-DLC film was about 70 cycles at room temperature. The durability of the more than 1.0-nm-thick P-CVD-DLC films increased to over 100 cycles at 100 °C. Moreover, at 200 °C, the durability of the 0.3–0.8-nm-thick P-CVD-DLC films, evaluated by cluster analysis, increased to nearly 100 or more cycles. At 200 °C, the extremely thin (<1 nm) hydrogen-containing P-CVD-DLC films seemed to exhibit a decreased friction owing to tribochemical reactions, which produced lubricant, like a hydrocarbon layer [27,28,29]. Although the durability of the P-CVD-DLC films was lower than that of the FCVA-DLC films, the durability of the P-CVD-DLC films increased at 100 and 200 °C, when compared with that measured at room temperature. This indicates that the durability of the P-CVD-DLC films increased at a certain temperature.

### 3.2. Mechanism of Durability Change at High Temperature

#### 3.2.1. Friction Durability at Room Temperature after High Temperature Heating

**Figure 11:**
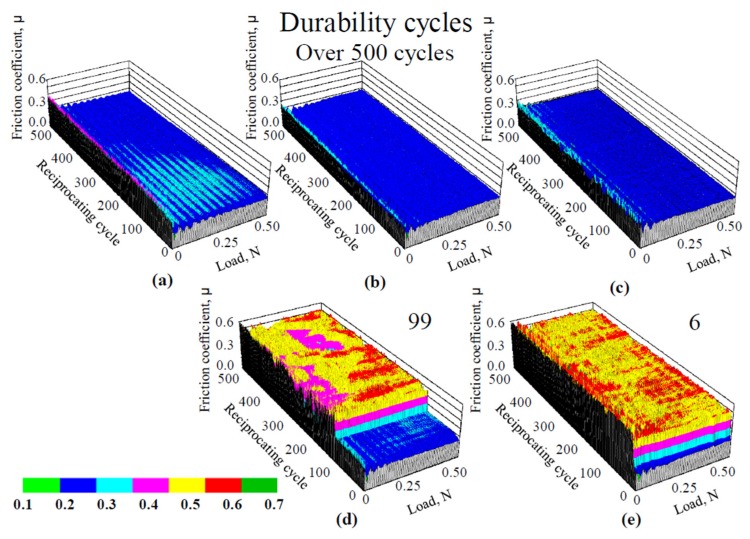
Friction coefficient of 1.0 nm thick FCVA-DLC film after heat treatment. (**a**) RT; (**b**) 100 °C; (**c**) 200 °C; (**d**) 300 °C; (**e**) 500 °C.

**Figure 12:**
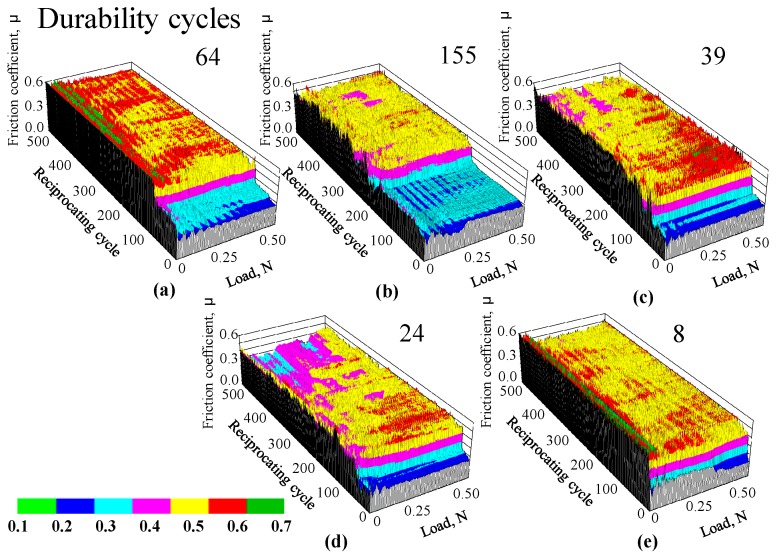
Friction coefficient of 1.0 nm thick ECR-CVD-DLC film after heat treatment. (**a**) RT; (**b**) 100 °C; (**c**) 200 °C; (**d**) 300 °C; (**e**) 500 °C.

**Figure 13:**
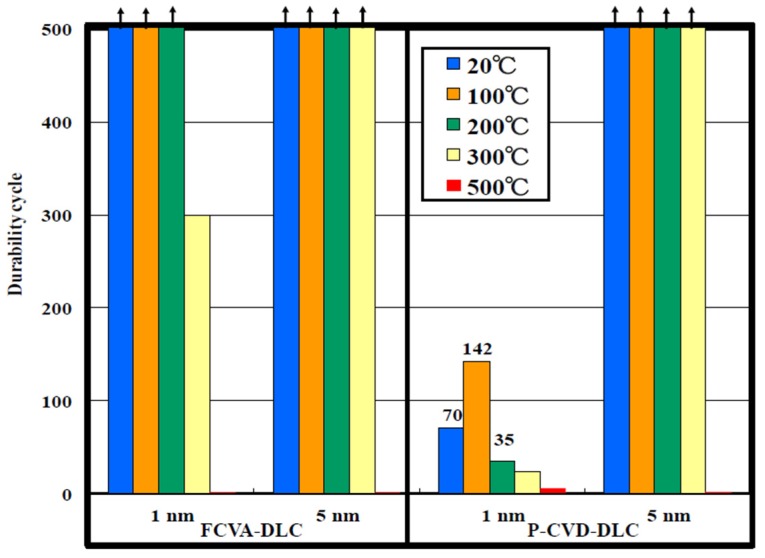
Durability cycles tested at room temperature after heating.

To clarify the influence of the high temperature heating on the friction properties of the films, we tested the films at room temperature after high temperature heating. The resulting friction coefficients measured for 1.0-nm-thick FCVA-DLC and P-CVD-DLC films, are shown in Figure 11 and Figure 12, respectively. Figure 13 shows the durability of 1- and 5-nm-thick DLC films after heating. The friction durability of the 1-nm-thick FCVA-DLC film, before heating and after heating to 100 and 200 °C, was superior to that of the 1-nm-thick P-CVD-DLC film and exceeded 500 cycles. At 300 °C, the durability decreased to 300 cycles. The 1-nm-thick P-CVD-DLC films endured a lower number of cycles than the FCVA-DLC films, at all pre-heating temperatures. The maximum cycle endurance was 142 cycles after pre-heating at 100 °C. The durability of the P-CVD-DLC films clearly decreased after heating at over 200 °C. In contrast, both the 5-nm-thick FCVA-DLC and P-CVD-DLC films endured over 500 cycles, both before heating and after heating to up to 300 °C. However, the durability of both types of film was very low at 500 °C. The heat resistance of the FCVA-DLC film was superior to that of the p-CVD-DLC film [12]. The dependence of the friction coefficient on the temperature of the films measured during and after heating, were obviously different. Therefore, the decrease in the durability of the FCVA-DLC film under high temperatures, shown in Figure 9, is deduced to not only have arisen from the degradation of the film at high temperatures, but also from a complex effect of the friction and high temperature. From the lower durability of the P-CVD-DLC films after high temperature heating, the improvement in the durability of the P-CVD-DLC film at high temperatures, shown in Figure 10, is deduced to originate from the proper curing of the film, and the probable formation of lubricant species through tribochemical reactions at high temperatures.

#### 3.2.2. Evaluation of Mechanism of Decrease and Increase in Durability of FCVA-DLC and P-CVD-DLC Films by Nano-Friction Test

##### 3.2.2.1. Nano-friction Test with and after High Temperature Heating

At room temperature, although the FCVA-DLC film was more durable than the P-CVD-DLC film, at high temperature, the friction experienced by the film increased and damage occurred rapidly. In contrast, the durability of the P-CVD-DLC film increased at high temperatures, while the durability of the FCVA-DLC film decreased. To clarify the mechanisms of these phenomena, we evaluated the nanotribological properties of the films, during and after heating.

**Figure 14:**
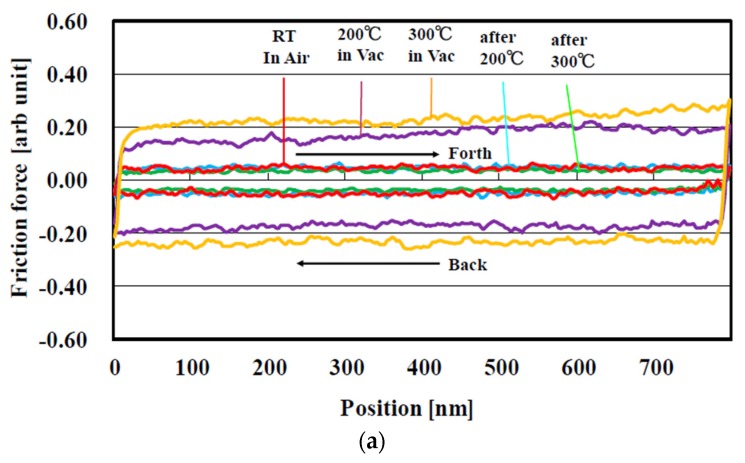

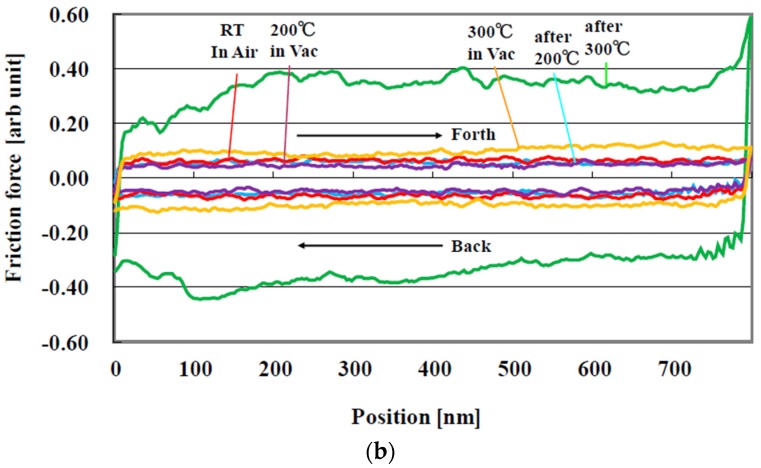
Friction force curve at and after RT, 200 and 300 °C. (**a**) FCVA-DLC film; (**b**) P-CVD-DLC film (Load 4500 nN).

The nano-friction force was evaluated from half of the difference between the forward and back curves measured for the FCVA-DLC film under a 4500-nN load, as shown in Figure 14a. The friction force measured at room temperature after heating (200 and 300 °C) hardly changed from that observed without heating. The friction force measured at 200 and 300 °C in a vacuum, was higher than those measured after heating. In contrast, the friction force measured for the P-CVD-DLC film at room temperature and 200 °C, and after heating at 200 °C, was low, but slightly increased at 300 °C, as shown in Figure 14b. However, the friction force increased remarkably after heating at 300 °C.

**Figure 15:**
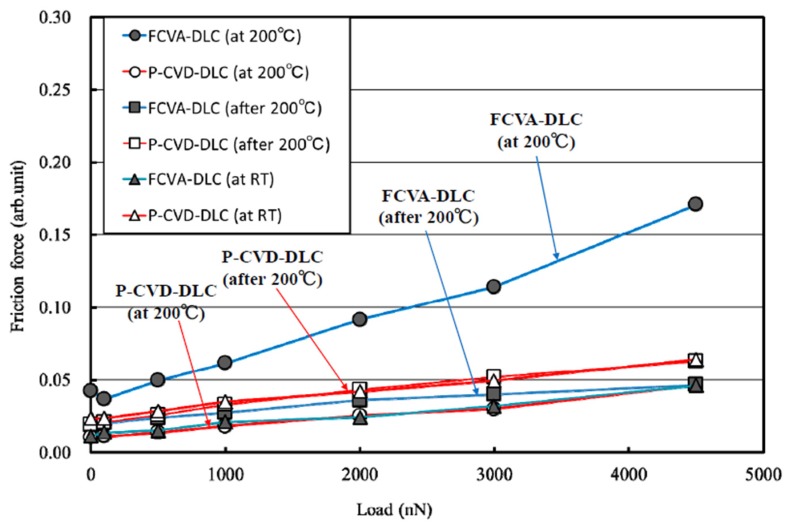
Nano-friction force dependence on load. (RT, 200 °C).

The dependence of the friction force on the load measured at room temperature and 200 °C, and that measured after heating to 200 °C, are shown in Figure 15. The friction force measured at room temperature after the FCVA-DLC film was heated to 200 °C was stable, and gradually increased with the load. Conversely, the friction force measured at 200 °C increased remarkably with the applied load. The friction coefficient also clearly increased. These results correspond to those of the macroscopic friction, shown in Figure 5. For the P-CVD-DLC films, the friction force measured after heating the sample at 200 °C, was relatively low, but increased exponentially with the load. In contrast, the friction force measured at 200 °C was low and increased in proportion with the load, as shown in Figure 15. At 4500 nN, the measured friction force was one-third of that measured after heating. In particular, the friction of this P-CVD-DLC film at 200 °C in a vacuum was the lowest measured under these test conditions. This reversal of the temperature dependence on the FCVA-DLC and P-CVD-DLC films was similar to that observed during the macroscopic friction tests.

**Figure 16:**
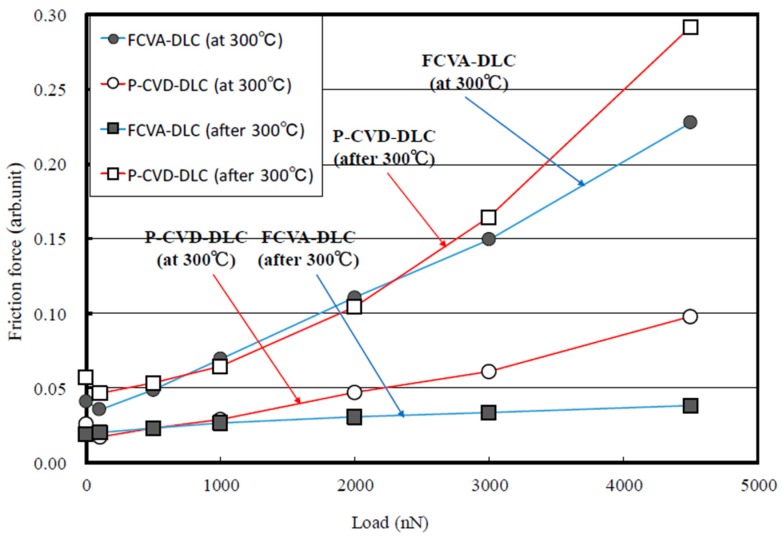
Nano-friction force dependence on load. (300 °C).

The dependence of the nano-friction force on the load at 300 °C, and after heating at 300 °C, is shown in Figure 16. Again, the FCVA-DLC and P-CVD-DLC films showed opposing tendencies. The friction force measured for the FCVA-DLC film after heating at 300 °C was low, and gradually increased with the load. In contrast, the friction force measured at 300 °C increased with the load, and became more than five times as high as that measured after heating at 4500 nN. Conversely, the friction force measured for the P-CVD-DLC film after heating at 300 °C was relatively high at a low load, and rapidly increased with the load, while that measured at 300 °C was relatively low, and increased proportionally with the load. At the 4500 nN load, the friction force measured at 300 °C was one-third of that measured after heating. The friction force measured for the FCVA-DLC film after heating was low, however, the friction force increased at high temperatures. Conversely, the friction force measured for the P-CVD-DLC film after heating was relatively high, and increased rapidly with the load, while that measured at high temperature was low and stable. This reversed temperature dependence of the FCVA-DLC and P-CVD-DLC films was similar to that observed during the macroscopic friction tests. These results contribute to the understanding of how durability depends on temperature in these systems.

##### 3.2.2.2. Decrease in Friction Durability of FCVA-DLC Films at High Temperature

The friction coefficient of the FCVA-DLC film was stable and low at room temperature after heating at less than 300 °C. This heat resistance was superior to that of the P-CVD-DLC film. In contrast, the friction coefficient measured at high-temperature increased gradually, and the durability of the film decreased. The decrease in friction durability can be considered as follows. For hydrogen-free FCVA DLC films, hydrocarbons, water, and so on, were adsorbed onto the surface of the films in the air. The adhesion of the FCVA-DLC film to its substrate was strong [21], so at room temperature, the residual DLC film and adsorbates were lubricous, and outstanding friction durability was obtained. In contrast, at high temperatures, the lubricous adsorbate was removed by the sliding, and the friction force increased. The adsorption onto the DLC affected the friction coefficient [30,31]. Then, because the FCVA-DLC films were hard and brittle, brittle fracture was induced at high temperatures by deformation of the substrate, owing to the complex action of the higher friction force and thermal stress. This caused the durability of the FCVA-DLC film to decrease. The wear abrasion produced by hard wear debris enhanced the damage of the film and increased its friction coefficient, similar to the behavior observed during the nanowear tests.

##### 3.2.2.3. Increase in Durability of P-CVD-DLC Films at High Temperature

For the hydrogen-containing P-CVD films, tribochemical hydrocarbon products that provide extremely low friction, were obtained as a result of the sliding in the vacuum [30,31]. Therefore, low nano-friction at high temperatures seemed to arise from the lubricating effect of these tribochemical reaction products. This is evidenced by the decrease in friction with increasing sliding cycles of the P-CVD-DLC films observed at 200 °C, shown in Figure 6b. At high temperatures, the tribochemical reactions progressed faster than at room temperature. Therefore, lubricous tribochemical reaction products from hydrogen-containing DLC films can cause their friction coefficient to decrease at high temperatures. The durability of the 0.3–0.8-nm-thick P-CVD-DLC films was greater at 200 °C, than at 100 °C. Therefore, it can be concluded that the tribochemical products in the P-CVD-DLC films reduced the friction, and increased the durability of the film at high temperatures. In contrast, the friction force tested after heating the films at 200 °C, was higher than that tested at 200 °C. The lower durability of the films after heating to 200–500 °C, is believed to have been caused by the degradation of the films, owing to the desorption of the hydrogen at the high temperatures, as evaluated by thermal desorption spectroscopy (TDS) [32]. The durability of the P-CVD-DLC film tested at room temperature after heating, was considerably lower than that of the FCVA-DLC film. However, the friction coefficient of the P-CVD-DLC films remained low for a greater number of durability test cycles at high temperatures. This decrease in the friction coefficient of the P-CVD-DLC film at high temperatures, likely originated from the lubricous layer produced by tribochemical reactions of the hydrogencontaining P-CVD-DLC film at high temperatures. This lubricous layer enhanced the durability of the P-CVD-DLC film.

## 4. Conclusions

The tribological properties of extremely thin FCVA and P-CVD DLC films at high temperature, and after high temperature heating, were evaluated.

(1) At room temperature, the durability of the FCVA-DLC films was superior to that of the P-CVD-DLC films. However, at high temperatures, the friction durability of the sub-2-nm-thick FCVA-DLC films decreased. The lubricating effect of the FCVA-DLC films was lost after fewer sliding cycles at high temperatures. The durability of the P-CVD-DLC films increased at high temperatures, when compared to that measured at room temperature. The durability of the P-CVD-DLC films increased at a certain temperature.
(2) The dependence of the friction coefficient on the load and number of reciprocating cycles at different temperatures, were evaluated by statistical cluster analysis. The dependence was classified into two clusters; cluster-I represented a low friction region in which the DLC film was effective, and cluster-II represented a high friction region where the lubricating effect of the DLC film was lost. The friction durability cycle and load could be evaluated from the boundary conditions of these clusters.
(3) The friction coefficient of the FCVA-DLC film was stable and low at room temperature, after heating at less than 300 °C. The decrease in the durability of the FCVA-DLC film at high temperatures, likely originated from a combination of the degradation of the film at high temperature, and the complex action of friction and high temperature. The increase in the friction coefficient of the FCVA-DLC film at high temperatures, was attributed to the removal of the lubricous adsorbate on its surface during sliding at high temperatures. The hard and brittle FCVA-DLC film then fractured, and hard wear debris enhanced the damage of the film and increased its friction, similar to that observed during the nanowear tests.
(4) The durability of the P-CVD-DLC film after heating was considerably lower than that of the FCVA-DLC film. However, the friction coefficient of the P-CVD-DLC films tended to stay low for more durability test cycles at high temperatures. This effect of the P-CVD-DLC film at high temperatures likely originated from the lubricous layer produced by the tribochemical reactions of the hydrogen-containing P-CVD-DLC film at high temperatures. This lubricous layer enhanced the durability of the P-CVD-DLC film.
(5) In our nano-friction tests, the friction coefficient dependence on temperature for FCVA-DLC and P-CVD-DLC films, showed opposing tendencies. The friction force measured for the FCVA-DLC film after heating was low. In contrast, the friction force increased at high temperatures. Conversely, the friction force measured for the P-CVD-DLC film after heating was relatively high, while that measured at high temperatures was low and stable. This reversed temperature dependence of the FCVA-DLC and P-CVD-DLC films was similar to that observed during the macroscopic friction tests. These results contribute to the understanding of how durability depends on temperature in these systems.

## Figures and Tables

**Table 1:** Average friction coefficient of cluster I and II.

Film	Average Friction Coefficient of Cluster-І	Average Friction Coefficient of Cluster- ІІ	Boundary Friction Coefficient
FCVA-DLC (RT)	0.132	---	---
FCVA-DLC (200 °C)	0.127	0.38	0.25
P-CVD-DLC (RT)	0.214	0.43	0.32
P-CVD-DLC (200 °C)	0.129	0.34	0.24
